# Knockdown of *SlYTHDF2* Accelerates Dark–Induced Tomato Leaf Senescence by Affecting the ABA Pathway

**DOI:** 10.3390/plants13192800

**Published:** 2024-10-06

**Authors:** Xinru Chen, Zihan Gao, Yangyang Li, Xiaoqian Nie, Qiaoli Xie, Guoping Chen, Zongli Hu

**Affiliations:** Laboratory of molecular biology of tomato, Bioengineering College, Chongqing University, Chongqing 400030, China; 13872655563@163.com (X.C.); liyangyang980207@163.com (Y.L.); 202319131134@stu.cqu.edu.cn (X.N.); qiaolixie@cqu.edu.cn (Q.X.); chenguoping@cqu.edu.cn (G.C.)

**Keywords:** *SlYTHDF2*, leaf senescence, ABA, dark induction

## Abstract

N6–methyladenosine (m^6^A) is a widespread post–transcriptional modification in eukaryotic mRNAs. Proteins with the YTH structural domain act as m^6^A–binding proteins by recognizing the m^6^A modification and regulating mRNA through this recognition. In this study, *SlYTHDF2*, a prototypical m^6^A –binding protein gene in the YTH family was expressed in various tissues, and subcellular localization analyses indicated that the SlYTHDF2 protein was localized in the nucleus and cytoplasm. *SlYTHDF2* knockout lines were obtained using CRISPR/Cas9 technology and showed the senesced leaves prematurely increased endogenous ABA accumulation compared with the wild type. Moreover, we found that dark promoted leaf senescence in *SlYTHDF2* knockout lines and exogenous ABA further accelerated leaf senescence under dark conditions. The qRT–PCR analysis revealed significant alterations in the expression of genes associated with the ABA pathway. Relative to the wild type, the *CR–slythdf2* plants exhibited reduced levels of photosynthetic pigments, higher accumulation of reactive oxygen species, and increased damage to cell membranes. Additionally, we discovered that SlYTHDF2 interacts with the chloroplast–binding protein SlRBCS3 through yeast two–hybrid and BiFC experiments. Overall, our data suggest the important role of *SlYTHDF2* in regulating tomato leaf senescence.

## 1. Introduction

The most prevalent internal modification of eukaryotic messenger RNAs is the methylation of the N^6^ nitrogen on adenosine, known as N^6^–methyladenosine (m^6^A). This modification is reversible and catalyzed by RNA methyltransferases and demethylases, and it is recognized by m^6^A –binding proteins [1,2]. Among these, m^6^A –binding proteins play a crucial role in determining the biological functions of m^6^A modifications, primarily influencing the translation status and stability of mRNAs. In mammals, several different types of m^6^A –binding proteins have been identified [3,4], including methyl–binding aromatic pockets (YTH structural domain) family proteins (YTHDF1, YTHDF2, YTHDF3, YTHDC1, and YTHDC2), insulin–like growth factor 2 mRNA–binding proteins (IGF2BPs), eukaryotic initiation factor 3 (eIF3), and heterogeneous nuclear ribonucleoprotein C (HNRNPC) [1,3,4,5,6], which play important functions in animal growth and development.

However, studies of m^6^A –binding proteins in plants are limited and have primarily focused on YTH domain–containing proteins. Research in plants has demonstrated that these proteins act as m^6^A –binding proteins, influencing various m^6^A –dependent developmental and growth processes in plants [7,8]. Mutations in *ECT2*, which contains the YTH domain, can accelerate the degradation of three transcripts associated with trichome morphogenesis, directly affecting trichome branching in *Arabidopsis thaliana* [9]. Additionally, the mutant of *ect2/ect3* exhibits significantly enhanced phenotypes, including slow leaf development and defective leaf morphology, which entirely depend on the intact m^6^A –binding sites of *ECT2* and *ECT3* [10,11]. There are also reports indicating that m^6^A –binding proteins play a role in the plant ABA pathway. In *Arabidopsis*, the deletion of *ECT8* results in the overactivation of ABA–responsive genes and an ABA hypersensitivity phenotype [12]. Moreover, *CPSF30–L* deletion extends the 3′UTR region of certain transcripts associated with phenotypes, accelerating their mRNA degradation and causing late flowering and ABA hypersensitivity [13,14]. In tomatoes, a total of nine genes encoding YTH–containing structural domains have been identified [15,16]. Knockdown of *SlYTH1* results in low seed germination, relatively short seedling root length, plant dwarfism, altered fruit shape, and a reduced number of ovaries [17]. Knockdown of *SlYTH2* leads to plant dwarfing, delayed internal fruit ripening, increased seed abortion, and heightened seedling sensitivity to ABA [18].

Leaf senescence represents a critical stage in the latter phases of plant growth and development, marked by a series of programmed degradation processes [19]. Accompanying the progression of leaf senescence, leaf cells undergo rather orderly changes in terms of cell structure and metabolism [20]. Initially, chloroplasts are the first organelles to degrade, with chlorophyll breakdown being a key aspect of leaf senescence. Concurrently, the degradation of chloroplast proteins commences early in senescence and is closely linked to chlorophyll degradation pathways [21,22]. Subsequently, leaf senescence is accompanied by enhanced reactive oxygen species metabolism and the resulting increase in membrane lipid peroxidation and membrane damage, leading to irreparable metabolic dysfunction and cell death [23]. Senescence involves changes in the expression of multiple genes, with several senescence–associated transcription factors such as *NAC*, *WRKY*, *MYB*, *C2H2 zinc finger*, *bZIP*, and *AP2/EREBP* families playing roles in dark–induced leaf senescence [24].

In addition, phytohormones play a crucial role in the process of plant senescence. Hormones such as abscisic acid, ethylene, and jasmonic acid promote senescence, whereas cytokinin and auxin inhibit this process [25,26,27,28,29]. Among them, abscisic acid, a sesquiterpenoid phytohormone, plays an important role in plant senescence. ABA content increases during leaf senescence, and exogenous application of ABA induces leaf senescence [19]. In rice, ABA has been shown to regulate NYCs (non–yellow coloring proteins) and D1 proteins, initiating chlorophyll degradation. Additionally, ABA binds to antioxidant responses, induces pre–transcriptionally NADPH oxidases, and regulates ROS generation by NADPH oxidases, thereby promoting leaf senescence [26]. In *Arabidopsis*, increased ABA levels induce the expression of *NAP*, and the *NAP* transcription factor activates the direct target gene, *SAG113* (encoding *PP2C*), which inhibits stomatal closure in leaves, accelerating water loss and thereby triggering the leaf senescence process [30,31]. Current data indicate that ABA plays a crucial role in promoting leaf senescence across a wide range of plants, suggesting its conserved role in this process and identifying it as a potential ideal target for regulating leaf senescence. Furthermore, prolonged darkness is a major inducer of leaf senescence, triggering significant physiological and genetic changes such as alterations in hormone dynamics, gene regulation, chloroplast integrity, and chlorophyll degradation [32].

Tomato (Solanum lycopersicum) is an important vegetable and cash crop with high nutritional and value advantages. As a result of its relatively small genome, short culture period, and mature genetic transformation system, tomato has become another model plant for studying gene function, in addition to *Arabidopsis thaliana* and rice. In this study, we constructed a *SlYTHDF2* knockout vector, and *SlYTHDF2* knockout lines were produced by *Agrobacterium*–mediated genetic transformation using CRISPR/Cas9 technology. The *SlYTHDF2* knockout lines exhibited a phenotype of premature leaf senescence, especially enhanced senescence of isolated leaves under darkness–induced senescence. In addition, endogenous ABA accumulation was increased in the knockout mutant, and exogenous ABA further promoted dark–induced senescence of isolated leaves in the mutant. This study lays a foundation for further elucidation of the biological functions of *SlYTHDF2* in tomato leaf senescence.

## 2. Results

### 2.1. Bioinformatics Analysis, Expression Pattern, and Subcellular Localization of SlYTHDF2

**Figure 1:**
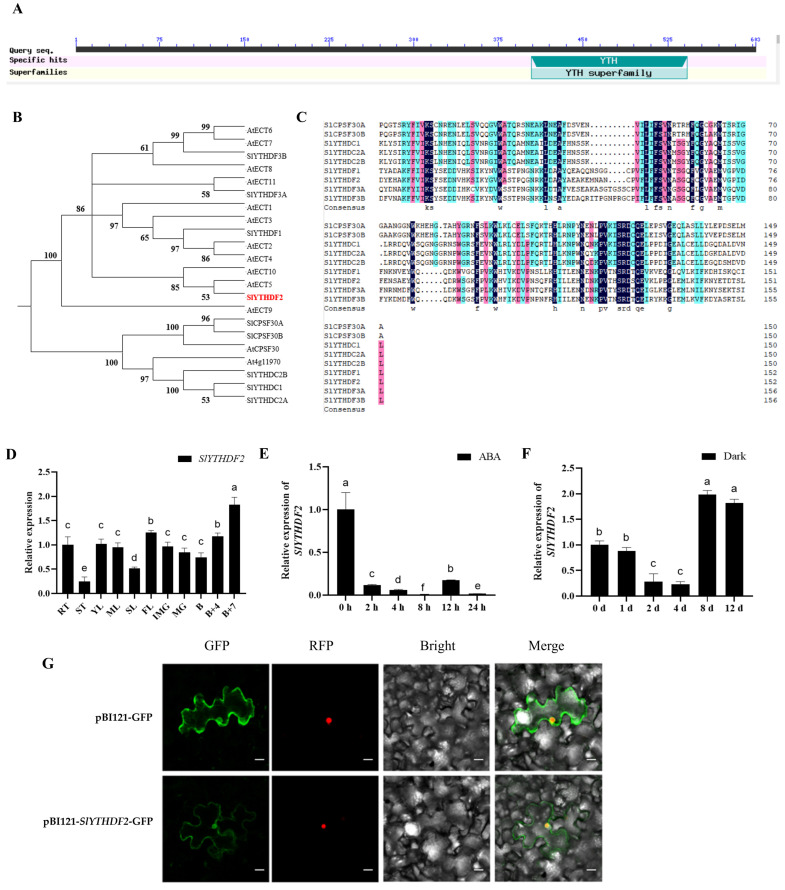
Bioinformatics analysis, expression pattern, and subcellular localization of *SlYTHDF2*. (**A**) Prediction of the conserved domain of SlYTHDF2 protein. (**B**) Phylogenetic analysis of YTH family proteins from *Solanum lycopersicum* (Sl) and *Arabidopsis thaliana* (At). The accession numbers are as follows: SlCPSF30A (XP_004231555.1), SlCPSF30B(XP_025885120.1), SlYTHDC1 (XP_004244593.1), SlYTHDC2A (XP_010324619.1), SlYTHDC2B (XP_004244595.1), SlYTHDF1 (XP_004228948.1), SlYTHDF2 (XP_004239610.1), SlYTHDF3A (XP_010314509.1), SlYTHDF3B (XP_004252938.1), AtECT1 (NP_001030629.1), AtECT2 (NP_001030690.1), AtECT3 (NP_851236.1), AtECT4 (NP_001321789.1), AtECT5 (NP_187912.2), AtECT6 (NP_001327579.1), AtECT7 (NP_001117446.1), AtECT8 (NP_001321639.1), AtECT9 (NP_001322517.1), AtECT10 (NP_200627.2), AtECT11 (NP_172452.3), AtCPSF30 (NP_174334.2), At4g11970 (NP_001328272.1). (**C**) Amino acid sequence alignment of SlYTHDF2 and other YTH proteins. (**D**) Quantitative RT–PCR analysis of the expression levels of the *SlYTHDF2* gene in roots (RT), stems (ST), young leaves (YL), mature leaves (ML), senescent leaves (SL), sepals (SE), flowers (FL), and fruits (pericarp) at immature green (IMG), mature green (MG), breaker (B), B + 4, and B + 7 stages. (**E**) Expression patterns of *SlYTHDF2* in leaves under the ABA treatments. (**F**) Expression patterns of *SlYTHDF2* in leaves under the dark–induced treatments. (**G**) Subcellular localization assay of SlYTHDF2 protein. GFP: green fluorescent protein; RFP: red fluorescent protein. Red fluorescent protein is used to locate the nucleus. Scale bar = 20 µm. Data are means ± SD of 3 biological replicates. Significant differences between samples are indicated according to Tukey’s method utilizing lower case letters (*p* < 0.05).

The *SlYTHDF2* gene is located on chromosome 5 and has an 1815 bp open reading frame (ORF) encoding a total of 604 amino acids. The conserved structural domains of the SlYTHDF2 protein were analyzed using CD Search in the National Center of Biotechnology Information (NCBI) database, and the results showed that SlYTHDF2 contains a YTH structural domain (Figure 1A). Phylogenetic tree analysis showed that, similar to *Arabidopsis*, YTH proteins of tomato can be divided into two branches: the YTHDF subfamily and the YTHDC1 subfamily. Among them, tomato SlYTHDF2 belongs to the YTHDF subfamily and is more homologous to AtECT5 and AtECT10. (Figure 1B). Multiple protein sequence alignments analysis of nine YTH proteins from tomato revealed highly conserved amino acid sequences within the YTH structural domains (Figure 1C).

To investigate the potential function of *SlYTHDF2* in the growth and development, the gene expression levels of *SlYTHDF2* in various tissues during tomato growth and development were quantitatively analyzed using qRT–PCR. The results indicated that *SlYTHDF2* was expressed in all tissues of WT (wild–type) tomato, suggesting its involvement in multiple processes of tomato growth and development, highlighting its significant biological functions (Figure 1D). The expression of *SlYTHDF2* was notably down–regulated after 2 h of ABA treatment and remained low expression for 24 h (Figure 1E), suggesting that *SlYTHDF2* can respond to ABA. In subsequent experiments, the plants were subjected to dark –induction to assess the gene’s responsiveness. The results demonstrated that the expression levels of *SlYTHDF2* gradually decreased during the pre–dark–induction period (0–4 days), whereas in the post–dark–induction period (8 and 12 days), its expression was significantly up–regulated (Figure 1F). Therefore, prolonged dark treatment significant induced *SlYTHDF2* expression, indicating its respond to dark –induction.

To investigate the subcellular localization of SlYTHDF2 protein, a 35S::*SlYTHDF2*–GFP and 35S::GFP vector were constructed and co–injected with the nuclear localization vector for transient expression in tobacco leaves. After three days of cultivation, the leaves were collected for observation under a laser confocal microscope. The results displayed that the green fluorescence emitted by the 35S::GFP empty vector was uniformly distributed in the epidermal cells of tobacco leaves (Figure 1G). Similarly, the fluorescent signal from the 35S::*SlYTHDF2*–GFP fusion expression vector was distributed throughout the cells (Figure 1G). These observations imply that the tomato SlYTHDF2 protein localizes to both the nucleus and cytoplasm.

### 2.2. Knockdown of SlYTHDF2 Accelerates Tomato Leaf Senescence

**Figure 2:**
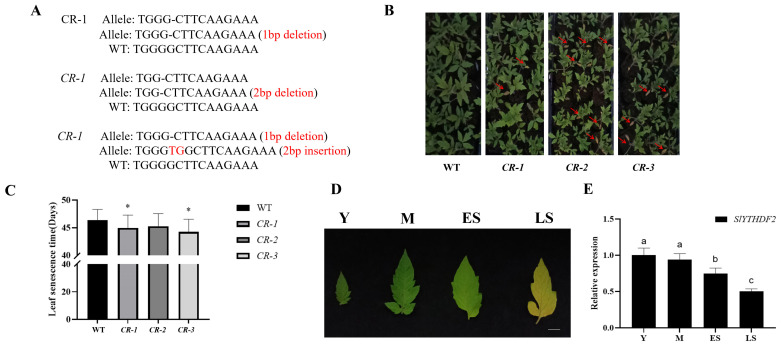
Phenotypic analysis of *CR–slythdf2* in the natural state of senescence. (**A**) The knockout types of *CR–slythdf2*. (**B**) Senescence phenotypes of WT and S *CR–slythdf2* plants. Red arrows indicate senescent leaves. (**C**) Statistical analysis of leaf senescence time at different periods for WT and *CR–slythdf2* plants (Statistically significant differences were determined using Student’s *t*–test; * *p* < 0.05). (**D**) Different developmental stages of WT leaves, Y (young leaves), M (mature leaves), ES (early senescent leaves), and LS (late senescent leaves). Bar, 1 cm. (**E**) Expression levels of *SlYTHDF2* in WT leaves at different developmental stages. Data are means ± SD of 3 biological replicates. Significant differences between samples were indicated according to Tukey’s method utilizing lower case letters (*p* < 0.05).

To explore the biological function of *SlYTHDF2*, the CRISPR/Cas9 gene editing technology was used to construct knockout vectors. Subsequently, we successfully generated three knockout transgenic lines, designated *CR–1*, *CR–2*, and *CR–3*. *CR–1* had a one–base deletion, *CR–2* had a two–base deletion, and *CR–3* had a one–base deletion on one strand and a two–base insertion on the other (Figure 2A). All three mutations resulted in frameshift mutations in the gene, and likely disrupted the function of SlYTHDF2 protein. Later observations showed that the first leaf of the *SlYTHDF2* knockout lines senesced earlier compared to the WT. Specifically, the leaves of the *CR–slythdf2* lines had just begun to yellow when the leaves of the WT had not yet shown significant yellowing. (Figure 2B). The first leaves of the WT exhibited senescence at 46.3 days, whereas the first leaves of the *SlYTHDF2* knockout lines *CR–1*, *CR–2*, and *CR–3* senesced at 44.9, 45.2, and 44.2 days, respectively (Figure 2C). To further elucidate the role of *SlYTHDF2* in tomato leaf senescence, we investigated its expression levels in young, mature, early senescent, and late senescent tomato leaves (Figure 2D). The results showed that the expression level of *SlYTHDF2* gradually decreased with increasing leaf senescence (Figure 2E). These results indicate that *SlYTHDF2* knockdown is able to promote tomato leaf senescence in the natural state.

### 2.3. Knockdown of SlYTHDF2 Accelerates Dark–Induced Senescence in Isolated Leaves

**Figure 3:**
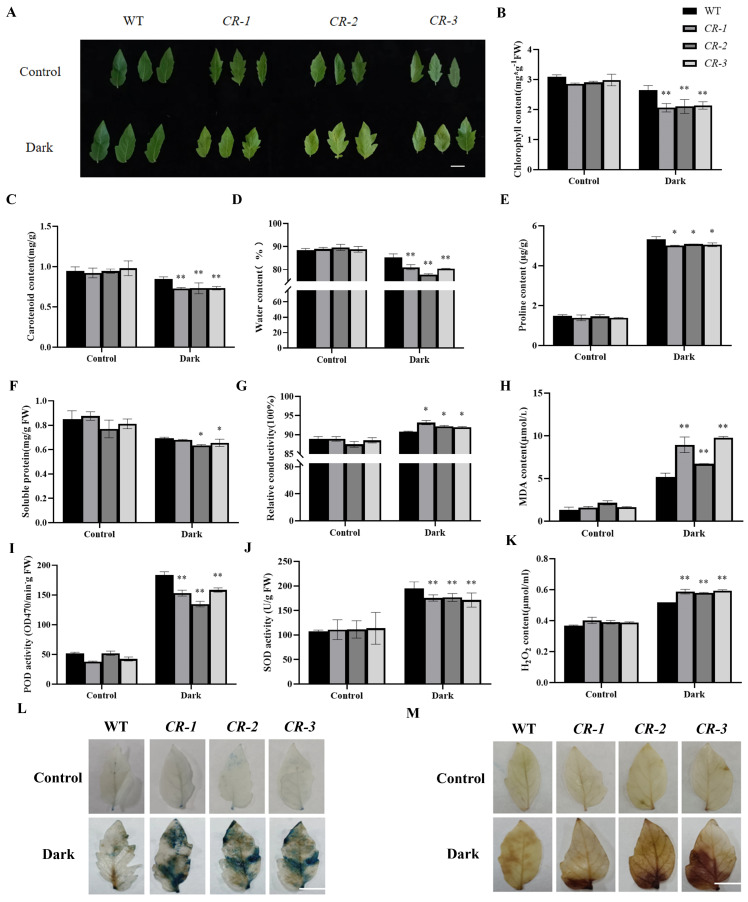
Phenotypic and physiological characteristics of *CR–slythdf2* and WT plants during dark–induced senescence. (**A**) Leaves senescence status of WT and *CR–slythdf2* plants in dark –induction. Bar, 1 cm. **B**–**K**: Chlorophyll content (**B**), carotenoid content (**C**), water content (**D**), proline content (**E**), soluble protein content (**F**), relative conductivity (**G**), MDA content (**H**), POD content (**I**), SOD content (**J**), H_2_O_2_ content (**K**). (**L**,**M**) Trypan blue and DAB staining. Data are means ± SD of 3 biological replicates. Statistically significant differences were determined using Student’s *t*–test; * *p* < 0.05, ** *p* < 0.01.

Since *SlYTHDF2* expression was notably up–regulated under prolonged dark conditions (Figure 1F), we conducted dark induction experiments on isolated leaves from both WT and *CR–slythdf2*. After 11 days of dark induction, leaves from the *CR–slythdf2* exhibited a clear wilting and yellowing phenotype, whereas WT leaves had not yet shown noticeable yellowing or signs of senescence (Figure 3A). Further investigations revealed significantly lower chlorophyll (Figure 3B) and carotenoid levels (Figure 3C) in isolated leaves of the *CR–slythdf2* compared to WT after 11 days of dark induction. Additionally, the water content of the *SlYTHDF2* knockout lines were notably lower than that of WT (Figure 3D). These findings suggested that isolated leaves of *CR–slythdf2* exhibit enhanced senescence by accelerated degradation of photosynthetic pigments along with reduced water retention capacity in dark induction.

To further determine the extent of damage in *CR–slythdf2* leaves under dark–induced conditions, the proline and soluble protein contents of treated isolated leaves were examined. The results showed that the proline content (Figure 3E) and soluble protein content (Figure 3F) in the isolated leaves of the *CR–slythdf2* plants were significantly lower than those of the WT. And the relative conductivity (Figure 3G) and MDA (Malondialdehyde) (Figure 3H) content of *CR–slythdf2* leaves were significantly higher compared to the WT. These findings indicate that cells in tomato leaves of *CR–slythdf2* were more severely damaged under dark conditions. Moreover, both (POD) peroxidase (Figure 3I) and SOD (Superoxide dismutase) activities (Figure 3J) were significantly lower in isolated leaves of the *CR–slythdf2* compared to WT. Conversely, the H_2_O_2_ content (Figure 3K) was significantly higher in isolated leaves of *SlYTHDF2* knockout lines compared to the WT. Trypan blue staining also showed more dead cells in the leaves of *CR–slythdf2* plants than WT after 11 days of dark induction (Figure 3L). DAB (3,3–N–diaminobenzidine tertrahydrochloride) staining further revealed that the brown areas were larger in the *CR–slythdf2* than WT (Figure 3M). These results indicate the reduced scavenging capacity of reactive oxygen species induced by darkness in isolated leaves of *CR–slythdf2*. This exacerbation contributed to heightened cellular damage and consequently accelerated leaf senescence.

### 2.4. Knockdown of SlYTHDF2 Lines Results in Increased Endogenous ABA Accumulation and Enhanced Sensitivity to Exogenous ABA

**Figure 4:**
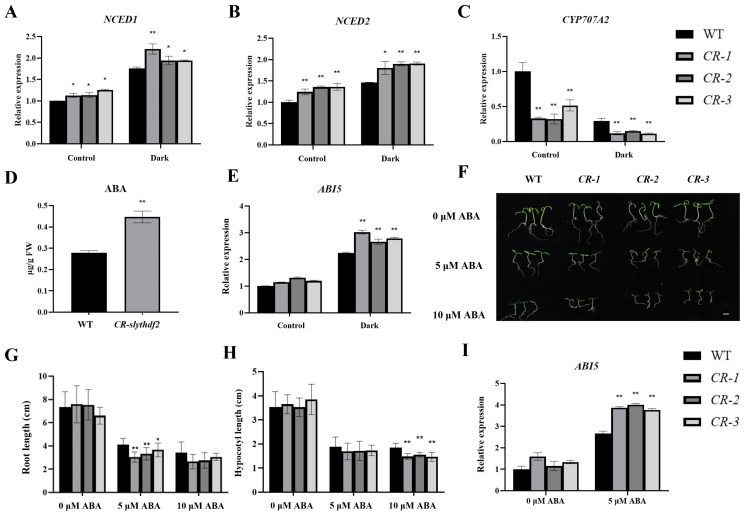
Analysis of endogenous ABA content and sensitivity to exogenous ABA in WT and *CR–slythdf2* plants. Expression levels of genes related to ABA synthesis and degradation, including *NCED1* (**A**), *NCED2* (**B**), and *CYP707A2* (**C**) before and after dark treatment (3 days). (**D**) Endogenous ABA content leaves of WT and *CR–slythdf2* plants. (**E**) Expression levels of *ABI5* related to ABA responsive before and after dark treatment. (**F**) Comparison of ABA sensitivity between WT and *CR–slythdf2* seedlings. Bar, 1 cm. (**G**) Root length. (**H**) Hypocotyl length. (**I**) Expression levels of *ABI5* before and after ABA treatment. Data are means ± SD of 3 biological replicates. Statistically significant differences were determined using Student’s *t*–test; * *p* < 0.05, ** *p* < 0.01.

Given that the expression levels of *SlYTHDF2* were significantly down–regulated under exogenous ABA induction (Figure 1E), we hypothesized that *SlYTHDF2* may influence the regulation of ABA pathways. Using qRT–PCR assay, we observed that the expression levels of *NCED1* and *NCED2* (genes related to ABA synthesis [33]) were significantly higher in *CR–slythdf2* plants compared to the WT plants (Figure 4A,B), and the expression levels of *CYP707A2* (genes related to ABA degradation [34]) were substantially lower in *CR–slythdf2* plants (Figure 4C), both before and after dark induction. These findings suggest that the *CR–slythdf2* plants led to increased expression of ABA–synthesizing genes and reduced expression of ABA–degrading genes. To further validate these results, we conducted an experiment to measure endogenous ABA content and observed that the ABA content in *CR–slythdf2* plants was significantly higher compared to the WT (Figure 4D). Moreover, the expression levels of *ABI5* (ABA responsive gene [35]) in *CR–slythdf2* plants were not significantly different from the WT plants before dark induction, whereas the expression levels of *ABI5* in *SlYTHDF2* knockdown lines were significantly higher after 3 days of dark induction (Figure 4E). In subsequent experiments, the plants were subjected to exogenous ABA to verify the extent of the *SlYTHDF2* gene response to ABA (Figure 4F). The results demonstrated that *CR–slythdf2* plants exhibited inhibition of root length and hypocotyl growth compared to the WT (Figure 4G,H). Correspondingly, after 5 μM ABA treatment, the expression levels of the ABA response factor *ABI5* were significantly higher in *CR–slythdf2* seedlings than in the WT (Figure 4I). These results suggest that the *SlYTHDF2* knockout lines exhibit higher sensitivity to ABA, indicating that *SlYTHDF2* may affect ABA pathway in plants.

### 2.5. ABA Further Accelerates Senescence of SlYTHDF2 –Knockout Leaves under Dark Conditions

**Figure 5:**
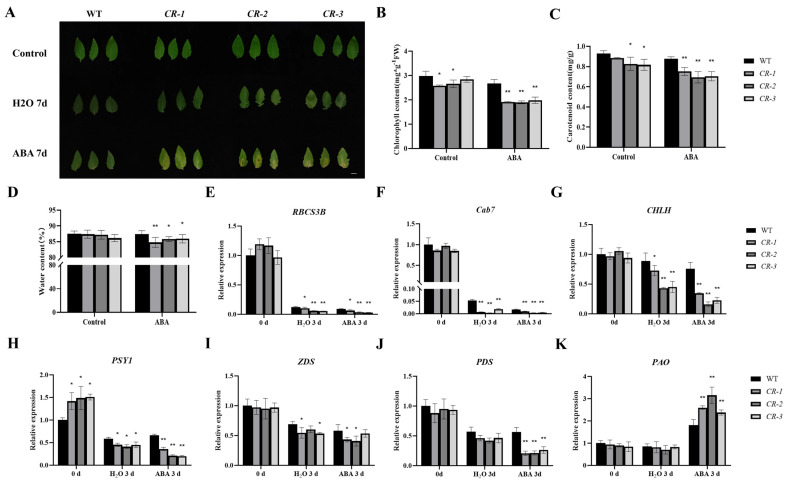
Leaves changes in *CR–slythdf2* and WT after ABA–induced senescence treatment. (**A**) Senescence phenotypes of leaves of WT and *SlYTHDF2* knockout lines after 100μM ABA in dark induction for 7 days. (**B**–**D**) Chlorophyll content (**B**), carotenoid content (**C**), water content (**D**). qRT–PCR was used to measure the expression levels of genes related to photosynthesis–related genes photosynthesis, chlorophyll synthesis, carotenoid syn–thesis, and chlorophyll degradation in *CR–slythdf2* and WT leaves, including *RBCS3B* (**E**), *Cab7* (**F**), *CHLH* (**G**), *PSY1* (**H**), *ZDS* (**I**), *PDS* (**J**), and *PAO* (**K**). Data are means ± SD of 3 biological replicates. Statistically significant differences were determined using Student’s *t*–test; * *p* < 0.05, ** *p* < 0.01.

To further assess the role of *SlYTHDF2* in ABA response in tomatoes, we treated leaves of *CR–slythdf2* plants with exogenous ABA during dark–induced leaf senescence experiments, using water as control. The results revealed that leaves isolated from *SlYTHDF2* knockout lines exhibited significantly more yellowing after 7 days of ABA treatment compared to the WT (Figure 5A). *CR–slythdf2* plants showed significantly more senescence following ABA treatment compared to water treatment (Figure 5A). After ABA induction, the chlorophyll content (Figure 5B), carotenoid content (Figure 5C), and natural water content (Figure 5D) were significantly lower in the isolated leaves of *SlYTHDF2* knockout lines compared to those of the WT. The qRT–PCR results revealed that the expression levels of genes related to photosynthesis (*RBCS3B* and *Cab7*), chlorophyll synthesis (*CHLH*), and carotenoid synthesis (*PSY1*, *ZDS*, and *PDS*) were significantly decreased in ABA–treated *CR–slythdf2* leaves compared with water treatment (Figure 5E–J), whereas the expression of genes related to chlorophyll degradation (*PAO*) was significantly increased (Figure 5K). These results indicate that ABA induces a reduction in chlorophyll synthesis and accelerates the degradation of chlorophyll in isolated leaves of *SlYTHDF2* knockout lines, resulting in leaves exhibiting an earlier yellowing phenotype.

**Figure 6:**
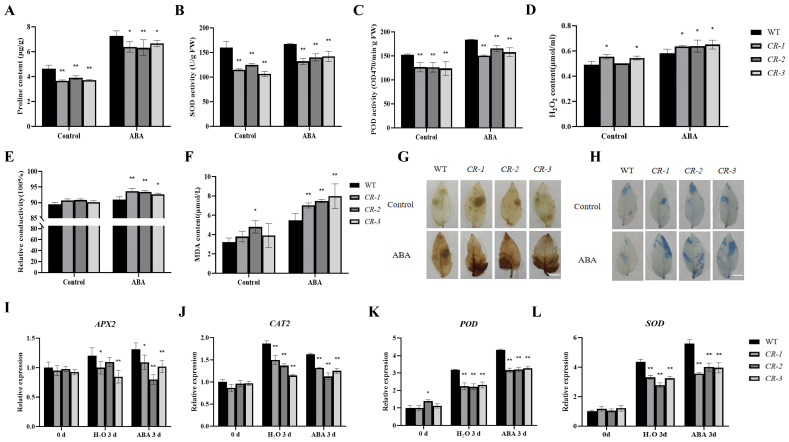
Comparison of cell damage indicators, ROS content between WT and *CR–slythdf2* lines after ABA treatment. (**A**–**F**) Physiological parameters of leaves of WT and *SlYTHDF2* knockout lines after 100μM ABA in the dark induction for 7 days, including proline content (**A**), SOD activity (**B**), POD activity (**C**), H_2_O_2_ content (**D**), relative conductivity (**E**), MDA content (**F**), DAB staining (**G**), trypan blue staining (**H**). The expression levels of reactive oxygen scavenging–related enzyme genes, including *APX2* (**I**), *CAT2* (**J**), *POD* (**K**), and *SOD* (**L**). Data are means ± SD of 3 biological replicates. Statistically significant differences were determined using Student’s *t*–test; * *p* < 0.05, ** *p* < 0.01.

To validate the role of *SlYTHDF2* in ABA–induced leaves senescence, we examined physiological parameters related to cell damage in isolated leaves of WT and *CR–slythdf2* plants. The results revealed that after 7 days of ABA induction, the proline content, SOD activity, and POD activity were significantly lower in isolated leaves of *SlYTHDF2* knockout lines compared to the water treatment (Figure 6A–C). In addition, the H_2_O_2_ content, relative conductivity, and MDA content were significantly higher in the WT (Figure 6D–F). DAB staining revealed that after 7 days of ABA induction, the brown staining areas were significantly larger and darker in isolated leaves of *CR–slythdf2* plants compared to water treatment (Figure 6G). Similarly, trypan blue staining indicated significantly larger blue staining areas in isolated leaves of *SlYTHDF2* knockout lines after 7 days of ABA induction compared to water treatment (Figure 6H). Furthermore, the expression levels of reactive oxygen scavenging–related enzyme genes (*APX2*, *CAT2*, *POD*, and *SOD*) were significantly down–regulated in isolated leaves of *CR–slythdf2* plants following ABA induction compared to water treatment (Figure 6I–L). These results suggest that ABA–induced reduction in osmoregulatory substance content and inhibition of reactive oxygen species scavenging pathways in *CR–slythdf2* resulted in elevated reactive oxygen species levels, increased membrane lipid peroxidation, and cellular damage, thereby accelerating leaf senescence. Collectively, these findings further underscore the role of *SlYTHD2* in influencing dark–induced leaf senescence via the ABA pathway.

### 2.6. SlYTHDF2 Interacts with SlRBCS3

**Figure 7:**
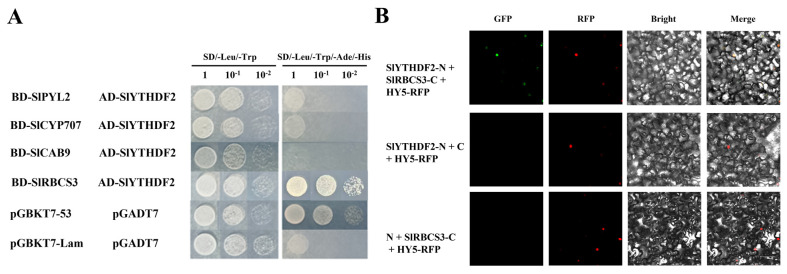
SlYTHDF2 interacts with SlRBCS3. (**A**) Yeast two––hybrid assays were used to examine the protein––protein interactions between SlYTHDF2 and SlPYL2, SlCYP707, SlCAB9, and SlRBCS3. Positive control: pGBKT7–53 + pGADT7–T; negative control: pGBKT7–Lam + pGADT7–T. (**B**) Interaction of SlYTHDF2 with SlRBCS3 by bimolecular fluorescence complementation assays. HY5–RFP, a nuclear localization control. RFP, red fluorescent protein; YFP, yellow fluorescent protein. Scale bars, 50 μm.

To further explore the molecular mechanism of *SlYTHDF2* in isolated leaves senescence, we attempted yeast two–hybrid experiments with proteins that may interact with SlYTHDF2. Based on the findings that ABA plays a crucial role in promoting dark–induced senescence of isolated leaves from *CR–slythdf2* plants, we conducted reciprocal experiments screening the ABA–responsive components PYL2 and CYP707, a key protein in the ABA degradation pathway [36,37,38]. Results from yeast two–hybridization demonstrated that neither PYL2 nor CYP707 interacted with SlYTHDF2. Moreover, yeast two–hybrid experiments were conducted on two proteins, CAB9 and RBCS3, which were screened for their role in photosynthesis [39,40]. Yeast two–hybridization results indicated that yeast cells co–expressing SlYTHDF2–AD and SlRBCS3–BD were able to grow on a four–deficient medium (SD/–Leu–Trp–His–Ade), similar to yeast cells containing the positive control pGADT7–T and pGBKT7–53 (Figure 7A). To test the in vivo interaction of SlYTHDF2 with SlRBCS3, bimolecular fluorescence complementation (BiFC) assays were used. By fusing SlYTHDF2 to the N–terminus of yellow fluorescent protein (nYFP), SlYTHDF2–nYFP was generated, and SlRBCS3 was fused to the C–terminus of YFP (cYFP) to generate SlRBCS3–cYFP. The results showed that stronger fluorescent signals were detected in the epidermal cell nucleus of *N. benthamiana* leaves only when SlYTHDF2–nYFP was co–expressed with SlRBCS3–cYFP (Figure 7B). These findings revealed the interaction of SlYTHDF2 with SlRBCS3 in vivo.

## 3. Discussion

### 3.1. SlYTHDF2 Accelerates Aging in Its Natural State

The m^6^A modification is a type of transcriptional regulation that plays a crucial role in various processes of plant growth and development [41,42]. Current studies have highlighted m^6^A’s involvement in diverse regulatory mechanisms. Among them, its major function is the regulation of RNA stability, primarily mediated by various m^6^A –binding proteins. YTHDF2, for instance, recruits the CCR4–NOT complex via interactions with its CNOT1 subunit, facilitating the de–adenylation and decay of m^6^A –containing RNA [43]. Similarly, *YTHDC1* promotes the decay of a subset of m^6^A –modified RNAs [44]. *FY* is the *Arabidopsis* homolog of the polyadenylation factor *Pfs2p* in yeast and *WDR33* in mammals. A mutation in *FY* disrupts the recognition of AAUAAA–like polyadenylation signals, thereby affecting mRNA stability [45]. It has been shown that dark induction leads to an increase in overall m^6^A levels and that the expression of m^6^A –binding proteins is strongly affected in the dark, and that senescence is enhanced in dark–induced senescence in *ect2/ect4* mutants [46]. In this study, we similarly observed that the first leaves senescence occurred earlier in *SlYTHDF2* knockout lines compared to the WT (Figure 2B).

### 3.2. SlYTHDF2 Further Accelerates Dark–Induced Leaf Senescence in Plants

A key external trigger of senescence is exposure to prolonged darkness, a process known as dark–induced senescence (DIS), and given the importance of leaves in photosynthesis, light deprivation or darkness can show significant senescence phenotypes in leaves, a process known as dark–induced leaf senescence (DILS) [47,48]. DILS has been used as a convenient model system for studying leaf senescence. We conducted dark–induced senescence experiments on isolated leaves, and the results demonstrated that the yellowing senescence phenotype appeared sooner in the *CR–slythdf2* plants than in the WT after DIS (Figure 3A). Plant senescence leads to water loss, ion leakage, reactive oxygen species (ROS) production, increased membrane fluidity, and lipid peroxidation in aging tissues [49]. In our study, physiological and biochemical assays indicated that *CR–slythdf2* plants exhibited lower levels of photosynthetic pigments and osmoregulatory substances in dark–induced isolated leaves compared to WT plants (Figure 3B–G). Consequently, this led to reduced H_2_O_2_ content and decreased activities of POD and SOD enzymes (Figure 3I–K). These conditions result in excessive accumulation of reactive oxygen species, intensifying cellular oxidative damage.

### 3.3. SlYTHDF2 Accelerates Dark–Induced Plant Leaf Senescence through the ABA Pathway

ABA is a crucial hormone that regulates plant growth, development, and responses to abiotic stresses like drought and high salinity. Previous studies have demonstrated that many genes involved in ABA synthesis (such as *AtNCED2* and *AtNCED3*), metabolism, or signaling undergo varying degrees of alteration during leaf senescence [25,33,50,51]. Studies have demonstrated that the deletion of certain m^6^A –binding proteins influences plant sensitivity to ABA, as well as the endogenous ABA content [13,14]. It has been shown that deletion of some m^6^A –binding proteins affects plant sensitivity to ABA as well as endogenous ABA content. For example, knockdown of CPSF30–L resulted in Arabidopsis seed germination and seedlings showing a hypersensitive phenotype to ABA. In the absence of *ECT8*, there is impaired segregation of m^6^A –modified *PYL7* transcripts in stress granules, leading to enhanced translation and cytoplasmic overaccumulation of PYL7 protein [12]. Therefore, we investigated the role of *SlYTHDF2* in the ABA signaling pathway. After dark treatment, the qRT–PCR results indicated that the expression levels of ABA–biosynthesis genes *NCED1* and *NCED2* were up–regulated in the *CR–slythdf2* and WT plants (Figure 4A,B), expression levels of ABA degradation *CYP707A2* were down–regulated (Figure 4C), and the ABA content in *CR–slythdf2* plants was significantly higher compared to the WT (Figure 4A–E). This suggests that the *SlYTHDF2* knockout lines may have influenced the endogenous ABA content. Exogenous application of ABA markedly inhibited root and hypocotyl elongation in the *SlYTHDF2* knockout lines (Figure 4F–H), highlighting their heightened sensitivity to ABA. Additionally, applying ABA to dark–induced isolated leaves further accelerated senescence compared to dark treatment alone in the *CR–slythdf2* plants (Figure 5A). Senescence–related physiological indices exhibited more pronounced changes under these conditions than with darkness alone. Our findings indicate that knockdown of *SlYTHDF2* influences the sensitivity of tomato plants to exogenous ABA and alters the accumulation of endogenous ABA, which accelerated dark–induced senescence in isolated leaves. Notably, we found that *CR–slythdf2* had more pronounced senescence under ABA treatment than in water treatment, and thus we hypothesize that *SlYTHDF2* could further influence senescence through the ABA pathway. Significantly, qRT–PCR assay showed that the expression of photosynthesis (*RBCS3B* and *Cab7*) and chlorophyll synthesis (*CHLH*))–related genes was significantly down–regulated in *SlYTHDF2* knockout lines induced by ABA (Figure 5E–G). This corresponds with previous studies indicating that senescence results in the down–regulation of photosynthesis genes such as ribulose bisphosphate carboxylase (*RBCS*) and chlorophyll a/b binding protein 1 (*Cab1*) [40]. Protein degradation is a crucial process during aging, with chloroplasts being among the first organelles to undergo degradation [21,52]. Chlorophyll degradation is one aspect of leaf senescence, and yellowing leaves are caused by chlorophyll degradation while carotenoids are relatively stable [53]. The hydrolysis of chloroplast proteins initiates early in senescence, and the degradation pathways of chlorophyll and chloroplast proteins are partly interconnected [54]. In our study, we discovered that SlYTHDF2 interacts with the CO_2_–fixing enzyme RBCS in tomatoes (Figure 7). These findings suggest that *SlYTHDF2* may regulate senescence in tomato plants by influencing the photosynthetic pathway. Although we did not identify relevant proteins in the ABA pathway that interact with SlYTHDF2, it is probable that *SlYTHDF2* regulates the ABA pathway through other modes of regulation, which would require verification through further experiments.

## 4. Materials and Methods

### 4.1. Bioinformatics Analysis

Sequences of *Arabidopsis thaliana* and *Solanum lycopersicum* YTH family proteins were obtained from the NCBI (National Center for Biotechnology Information) database. The conserved structures of these proteins were predicted and analyzed using CD–Search in the NCBI database. The phylogenetic tree of SlYTHDF2 proteins was constructed using DNAMAN, and a multiple sequence comparison of YTH proteins was generated using MEGA 5.0. Furthermore, DNAMAN 5.2.2 software was used for multiple sequence alignment between SlYTHDF2 and related proteins of its family.

### 4.2. Plant Materials

Wild–type tomato (*Solanum lycopersicum Mill cv. Ailsa Craig*, *AC++*), *CR–slythdf2* transgenic plants, and *Nicotiana benthamiana* were used in this study. Tomato growth conditions were 28 °C/16 h during the day and 18 °C/8 h at night; the light intensity was 250 µmol m^−2^s^−1^ and the relative humidity was 60%.

### 4.3. Subcellular Localization Analysis

We amplified the *SlYTHDF2* coding sequence (CDS) without a stop codon via PCR and recombined it into pBI121–GFP for expression in fusion with GFP. The constructed vector was transformed into *Agrobacterium tumefaciens* strain GV3101, and pBI121–SlYTHDF2–GFP and control pBI121–GFP were infiltrated into 4–week–old tobacco leaves by *Agrobacterium*–mediated genetic transformation. After *Agrobacterium* infiltration, the leaves were incubated in the dark for 2 days and then transferred to light for 2 days. The fluorescence signals were observed under a laser confocal microscope. One of the nuclear localization signals was the *HY5*–RFP fusion protein; *HY5* is a transcription factor, which has been reported in the literature to be localized in the nucleus [55]. The primers used to construct the vector are shown in Table S1.

### 4.4. Expression Pattern Analysis

Tissue expression pattern: Roots, stems, young leaves, mature leaves, senescent leaves, flowers (RT, ST, YL, ML, SL, FL), and fruit samples after IMG, MG, B, B + 4, and B + 7 stages of WT tomato were harvested. For the expression pattern under ABA treatment, 35–day–old WT tomato seedlings were sprayed with 100 μM ABA until the foliage was wet and then placed in an incubator; sampling was performed at 0 h, 2 h, 4 h, 8 h, 12 h, and 24 h post–treatment, with ddH_2_O (double distilled H_2_O) treatment serving as a control. To induce an expression pattern under darkness, 60–day–old WT plants were chosen. Leaves from the same section were placed in a glass dish lined with pre–moistened filter paper (3 mL of H_2_O). These leaves were kept in a dark environment at 22 °C, with additional 3 mL of H_2_O added dropwise every 3 days throughout the 12–day treatment period. Sampling was conducted at 0, 1, 2, 4, 8, and 12 days after the initiation of treatment.

### 4.5. SlYTHDF2 Knockout Vector Construction and Genetic Transformation

The knockout sites of the *SlYTHDF2* gene were designed using the knockout target design website CRISPR–P2.0 (http://crispr.hzau.edu.cn/CRISPR2/, accessed on 30 August 2024). The *CRISPR/Cas9–SlYTHD–F2* vector was constructed using the pKSE401 vector. This vector was transferred into *Agrobacterium* strain LBA4404, and the *SlYTHDF2* knockout lines were obtained using *Agrobacterium*–mediated genetic transformation of tomato. The *CR–slythdf2* sequence was then analyzed with sequencing technology to determine the knockout type. The primers used for vector construction are shown in Table S1.

### 4.6. Total RNA Extraction and Real–Time Fluorescence Quantitative PCR Analysis

Total RNA was extracted using RNAiso plus (TaKaRa Takara Bio Inc, Osaka, Japan), as described in previous studies. RNA was reverse transcribed to cDNA using M–MLV (TaKaRa) [56]. Transcript expression levels of specific genes were quantitatively analyzed using 2 × GoTaq^®^ qPCR Master Mix enzymes, using the tomato *SlYTHDF2* gene (*Solyc12g099090*) as an internal reference. Quantitative reverse transcription PCR (qRT–PCR) was conducted using the CFX Connect Real–Time System (Bio–Rad, South Granville, NSW, USA). The primers used for reverse transcription and qRT–PCR are shown in Table S1.

### 4.7. Darkness–Induced Leaf Senescence Experiment

Leaves from the same part and the same size of the WT and *CR–slythdf2* plants with uniform growth at 60 days were selected and placed in glass Petri dishes padded with filter paper, which was moistened with ddH_2_O and 50 μM ABA beforehand, and placed in a dark environment at 22 °C, where H_2_O and 50 μM ABA were added once every 3 days. Photographs were taken and samples were taken when the treatment group showed an obvious yellowing phenotype (about 11 days).

### 4.8. Measurement of Physiological Indicators Related to Photosynthetic Pigments

Chlorophyll content: Fresh leaves were ground to powder with liquid nitrogen, and chlorophyll was extracted in 80% ethanol until the precipitate was white, and the absorbance was measured at OD_646_ and OD_663_ to calculate the total chlorophyll content per gram of fresh weight. The formula was chlorophyll content (mg/g) = (20.29 × OD_646_ + 8.02 × OD_663_) × volume of extracted liquid (mL)/fresh weight of material (g) [57].

Total carotenoid content: Fresh leaves were ground to powder form with liquid nitrogen, and total carotenoids were extracted in a mixture of hexane/acetone = 3:2 until the precipitate was white, and the absorbance was measured at OD_450_ to calculate the total carotenoid content per gram of fresh weight. The formula was calculated as total carotenoids (mg/mL) = 4 × OD_450_ × volume of extracted liquid (mL)/[0.25 × 1000 × fresh weight of material (g)] [58].

### 4.9. Determination of Physiological Indicators Related to Water Content and Osmoregulatory Substances

Natural water content: The leaves were taken and weighed for the fresh weight Wf and then the leaves were wrapped in filter paper and placed in the oven, baked to a constant weight and then weighed for the dry weight Wd. The formula was calculated as follows: natural water content = (Wf − Wd)/Wf × 100%.

To determine the proline content, samples were treated as follows: First, the leaves materials were ground in liquid nitrogen. Then, 0.2 g of this ground material was mixed with 1.3 mL of 3% sulfosalicylic acid and heated in a boiling water bath for 10 min. After the boiling water bath and subsequent cooling, toluene was introduced to ensure complete reaction. Then, absorbance was measured at OD_520_ to construct a standard curve. The formula used to calculate proline content (μg/g) was proline content (μg/g) = (C × Vt)/(W × Vs) × 100%. W is the weight of the sample powder (g), C represents the proline content (μg/mL) derived from the proline standard curve, Vt is the volume of supernatant, and Vs is the volume of the sample used in the determination (mL) [59].

Determination of soluble protein content: The samples were mixed with a BSA standard solution, water, and Coomassie Brilliant Blue, and the absorbance was measured at OD_595_. A standard curve was then plotted. The following formula was applied: soluble protein content (mg/g) = (C × Vt)/(W × Vs) × 100%, where C is the soluble protein content (mg/g) in the sample tube from the standard curve; Vt is the total volume of the reaction solution (mL); W is the mass of the ground leaf (g); and Vs is the volume of the enzyme solution to be tested (mL) [60].

### 4.10. Measurement of Physiological Indices Related to Cell Damage

Determination of malondialdehyde content: The sample was ground in liquid nitrogen to powder; 5% TCA was added, 0.5% TBA was added to the supernatant, and then the absorbances at OD_450_, OD_532_, and OD_600_ were measured after a boiling water bath to calculate the malondialdehyde content of each gram of sample. The formula was calculated as MDA (μmol/L) = [6.45 × (OD_532_ − OD_600_) − 0.56 × OD_450_] × Vt (Vs × W), where Vt is the total volume of supernatant (mL); Vs is the volume of extract at the time of determination (mL); and W is the mass of ground leaves (g) [61].

Measurement of relative conductivity: The leaves were immersed in ddH2O for 12 h. At this time, the liquid conductivity (R1) was measured followed by a measurement of the liquid conductivity (R2) after a boiling water bath. These data were then used to calculate the relative conductivity. The following formula was applied: relative conductivity = (R1/ R2) × 100% [62].

H_2_O_2_ content: The sample was ground in liquid nitrogen until powdered, and the H_2_O_2_ content per gram of sample was calculated. The following formula was applied: H_2_O_2_ content (μm/g) = (C × Vt)/(W × Vs) × 100%, where C is the H_2_O_2_ content (μm/g) in the sample tube from the standard curve; Vt is the total volume of the reaction solution (mL); and W is the mass of the milled leaves (g) [63].

POD activity: The supernatant from the determination of soluble protein was taken and fully reacted with the reaction solution (PBS buffer, guaiacol, and H_2_O) and the absorbance values were measured at OD_470_ every 1 min for three times to calculate the POD activity per gram of samples. The formula was as follows: POD activity (OD_470_/min × g FW) = ΔA_470_ × Vt/ Vs/ W. The formula ΔA_470_ indicates the change in A_470_ per unit of time, and the meanings of Vt, Vs, and W are the same as those referred to in the determination of soluble protein content [37].

Detection of SOD activity: The supernatant was taken from the determination of soluble proteins and reacted fully with the reaction solution (PBS buffer, methionine, NBT, EDTA–Na_2_, FD, and H_2_O), and the absorbance value at OD_560_ was measured to calculate the SOD activity per gram of samples. The formula was calculated as SOD activity (U/g FW) = (ACK − AC) × Vt/(W × 0.5 × ACK × Vs), where ACK is the average value of A_560_ of the three control groups under light, AC is the A_560_ of the blank group under darkness, and the meanings of Vt, Vs, and W are the same as those referred to in the determination of soluble protein content [37].

### 4.11. DAB and Trypan Blue Staining Method

The staining steps were as follows: the leaves were placed in trypan blue staining solution, subjected to 50 rpm staining for 5 h, and then a boiling water bath for 10 min, and 95% ethanol decolorization was performed 2–3 times away from light until the leaves were completely decolorized. The leaves were placed in DAB staining solution, subjected to 50 rpm staining for 8 h, and 95% ethanol decolorization was performed 2–3 times away from light until the leaves were completely decolorized [64].

### 4.12. Yeast Two–Hybrid and BiFC Assays

Yeast two–hybrid assays: The bait plasmid pGBKT7– SlYTHDF2 and the prey plasmid pGADT7–SlRBCS3, pGADT7–SlCAB9, pGADT7–PYL2, and pGADT7–CYP707 were co-transformed into yeast strain Y2HGold. The transformed yeast was then plated on the SD medium lacking tryptophan and leucine followed by incubation for 3 days. Thereafter, a single colony was selected and inoculated on the supplemented SD medium devoid of tryptophan, histidine, adenine, and leucine. This plate was incubated upside down for 1–2 days [65]. BiFC assays: The recombinant SlYTHDF2–nYFP and SlRBCS3–cYFP vectors were transformed into the *Agrobacterium tumefaciens* GV3101. Then, SlYTHDF2–nYFP, SlRBCS3–cYF, and control HY5–RFP were infiltrated into 4–week–old tobacco leaves by *Agrobacterium*–mediated genetic transformation. The subsequent experimental steps refer to the Materials and Methods Section 4.3 Subcellular Localization Analysis [66]. The primers used to construct the vector are shown in Table S1.

### 4.13. Statistical Analysis

Statistical analysis was performed using one–way ANOVA and Student’s *t*–test. Data are expressed as mean ± SD (standard deviation). Data were analyzed using SPSS 26.0 software. All measurements were taken from the mean of at least three independent biological replicates [67].

## 5. Conclusions

In this study, based on the amino acid sequences of the mammalian m^6^A –binding protein HsYTHDF1 and the *Arabidopsis* m^6^A –binding proteins AtECT5 and AtECT10, we screened a potential m^6^A –binding protein SlYTHDF2 from tomato, constructed a *SlYTHDF2* knockdown vector using CRISPR/Cas9, and obtained three independent knockdown lines by *Agrobacterium*–mediated genetic transformation technology. In our study, knockdown of *SlYTHDF2* lines accelerated dark–induced senescence of isolated leaves, and *CR–slythdf2* lines resulted in up–regulation of abscisic acid synthesis, down–regulation of abscisic acid degradation–related genes, and increased accumulation of endogenous abscisic acid. Knockdown of *SlYTHDF2* lines resulted in enhanced sensitivity of seedlings to ABA and advanced senescence of isolated leaves under exogenous ABA–induced senescence, and SlYTHDF2 was found to be able to interact with SlRBCS3. Taken together, *SlYTHDF2* may regulate the senescence function of plant leaves under darkness–induced senescence through the ABA pathway as well as the photosynthetic pathway.

## Data Availability

Data are contained within the article or Supplementary Material.

## Supplementary Materials

The following supporting information can be downloaded at: https://www.mdpi.com/article/10.3390/plants13192800/s1, Table S1: All the primer sequences used in this study.
